# Effect of successful aging on mortality in older individuals: The PALA study

**DOI:** 10.1590/S1980-57642014DN82000015

**Published:** 2014

**Authors:** Ana Luiza Camozzato, Claudia Godinho, Márcia Lorena Fagundes Chaves1

**Affiliations:** 1 Dementia Clinic, Neurology Service, Hospital de Clínicas de Porto Alegre, RS, Brazil.; 2 UFRGS School of Medicine, Porto Alegre, RS, Brazil.; 3 Internal Medicine Department, UFRGS School of Medicine, Porto Alegre, RS, Brazil.; 4 Internal Medicine Department, UFCSPA School of Medicine, Porto Alegre, RS, Brazil.

**Keywords:** mortality rate, causes of death, successful aging, Brazil

## Abstract

The definition of successful aging and identification of predictors have been extensively reviewed, less attention however, has been given to the role of this condition on mortality.

**Objective:**

To evaluate the effect of aging status (normal or successful) on mortality in a South Brazilian population-based cohort, adjusted for sociodemographic and clinical variables, and to report the mortality rate and causes of death in this population.

**Methods:**

The baseline sample comprised 345 community-dwelling, independent and healthy Southern Brazilian older individuals who were followed for 12 years. Clinical, socio-demographic, functional and cognitive variables were assessed at baseline and during the follow-up. At baseline, 214 participants fulfilled criteria for successful aging, and 131 for normal aging. The main outcome was death.

**Results:**

The Cox regression model showed an increased risk for mortality in subjects with normal aging (HR=1.9; p=0.003) adjusted by age (HR=1.1; p<0.001) and by sex (HR=1.9; p=0.002). The overall mortality rate was 41% and the rate was significantly lower among successful than normal agers (p=0.001). The main causes of death were cardiovascular disease and cancer.

**Conclusion:**

Our main finding was an increased risk of mortality among normal in comparison with successful aging subjects, emphasizing the impact of the heterogeneity of the healthy aging process on mortality.

## Abstract (pt)

A definição de envelhecimento bem sucedido e a identificação dos seus preditores têm sido extensamente revisadas, entretanto o papel desta condição na mortalidade tem sido menos estudado.

**Objetivo:**

Avaliar o efeito do *status* de envelhecimento (normal ou bem sucedido) sobre a mortalidade em uma coorte de base populacional do Sul do Brasil, ajustado para variáveis sociodemográficas e clínicas. Descrever a taxa de mortalidade e as causas de morte nessa população.

**Métodos:**

A amostra inicial foi composta de 345 idosos residentes na comunidade, independentes e saudáveis, que foram acompanhados por 12 anos. Variáveis sociodemográficas, funcionais, cognitivas e clínicas foram avaliadas no início e durante o seguimento. No início do estudo, 214 participantes preencheram os critérios para envelhecimento bem-sucedido, e 131 para envelhecimento normal. O desfecho foi mortalidade.

**Resultados:**

O modelo de regressão de Cox mostrou um aumento do risco de mortalidade para indivíduos com envelhecimento normal (RC=1,9; p=0,003), ajustado para idade (RC=1,1; p<0,001) e sexo (RC=1,9; p=0,002). A taxa de mortalidade foi significativamente menor entre os idosos com envelhecimento bem sucedido em comparação aos idosos com envelhecimento normal (p=0,001). A taxa de mortalidade geral foi de 41%. As principais causas de morte foram doenças cardiovasculares e câncer.

**Conclusão:**

O principal resultado deste estudo foi o risco de mortalidade aumentado nos indivíduos com envelhecimento normal em comparação com os indivíduos com envelhecimento bem sucedido, enfatizando o impacto da heterogeneidade do processo de envelhecimento saudável sobre a mortalidade.

## INTRODUCTION

Mortality frequency rates in the elderly population can range from 9% to 98% according to different clinical and demographic sample characteristics and length of follow-up.^1,2^ The identification of risk factors for mortality in older subjects is relevant since efforts may be made not just to reduce this rate but especially to improve quality of life (reducing morbidity). Higher age has been described as the main risk factor for mortality in longitudinal population-based studies.^2-4^ Male sex,^3,5^ poor health status,^3,4^ depressive symptoms^2,3,6^ and functional disability have also been associated with mortality among older individuals.^3,7^

Clinical and mental diseases as well as functional disability are frequently observed with the aging process. However some individuals go further, with avoidance of severe disease and disability, enjoying healthy aging. Among this group, some older subjects go beyond, aging successfully without disability and with high cognitive, physical, and social functioning.^8-10^ The definition of successful aging and identification of predictors have been extensively reviewed;^8^ less attention however, has been given to the role of this condition in mortality. Our hypothesis was that mortality rates among "successful agers" are lower than those observed in the general older population. The aim of the present study was to evaluate the effect of successful aging on mortality in a population-based cohort of older individuals followed for 12 years, adjusted for sociodemographic and clinical variables. The mortality rate and causes of death for the whole of this sample were also evaluated.

## METHODS

The individuals included in the current study were derived from the Porto Alegre Longitudinal Aging (PALA) study,^11-13^ an ongoing longitudinal population-based study on aging and dementia started in 1996. At baseline, all subjects were healthy and independent, ≥ 60 years of age, and resided in the catchment area of the Hospital de Clinicas de Porto Alegre, in the city of Porto Alegre, southern Brazil. The exclusion criteria were medical conditions such as chronic renal disease, significant head injury, and stroke; psychiatric conditions such as major depressive disorder; uncorrectable vision or hearing loss, substance abuse or use of medications or other conditions that might impair cognition. This exclusion process was performed through application of a standardized protocol, including family and personal history-taking as well as neuropsychological and neurological evaluation. The final sample comprised 345 participants. The methods were published in more detail elsewhere.^11^ Demographic and medical data, parents' vital status, social support, engagement in leisure activities, the Montgomery-Asberg Depression Rating Scale (MADRS),^14^ Mini-Mental State Examination (MMSE),^15^ Clinical Dementia Rating (CDR),^16^ and the Activities of Daily Living (ADL)^17^ scale, were also assessed at baseline. All baseline instruments were re-applied at each follow-up assessment.

At baseline, successful aging was defined as very good state of health, a complete absence of functional disability and mood changes, and no cognitive impairment (MMSE score above the education-adjusted cutoff).^9,10^ A total of 214 participants fulfilled the successful aging criteria, representing 62% of the sample. Individuals who had age-determined responses and behaviors without contamination by specific disease processes were considered normal agers (N=131; 38%). These subjects showed typical non-pathologic age-linked losses, including minor health problems, a degree of cognitive frailty (lower MMSE scores), and some functional disability (lower scores on the Katz ADL scale).^12^ Baseline variables, such as aging status (categorized as normal or successful aging), age, sex, education, family income, age of parents at death, depressive symptoms, leisure activities and social support (measured by the presence of confidants) were analyzed as independent variables for subsequent mortality.

The vital status outcome (dependent variable) was assessed by annual phone calls from the research team to confirm which participants were still alive, and also thorough follow-up evaluation. Additionally, family members were required to contact the research team by phone when participants had died. Causes and dates of death were also obtained from official death-certificates. Individuals who completed the baseline evaluation and who were followed for 12 years or until their death, whichever occurred first, were included in the present study.

**Statistical analysis.** The ANOVA, Student's t-test and Chi-square association test were used for comparative and association analyses. The univariate Cox proportional hazard model was first used to estimate the relative risks and corresponding 95% CI of mortality in relation to baseline age, sex, education, family income, depressive symptoms, aging status, age of parent at death, depressive symptoms, leisure activities and social support. The multivariate Cox proportional hazards model was used to examine the independent effects of those variables that showed significant risk for mortality in the univariate model. All analyses were performed using the Statistical Package for the Social Sciences (SPSS, Version 17.0).

**Ethics procedures.** The study was approved by the Ethics Committee for Research of the Hospital de Clínicas de Porto Alegre. All participants and/or their proxies signed an informed consent form.

## RESULTS

The present sample comprised 234 older individuals. The drop-out rate over the 12-years follow-up was 30% (N=111). Table 1 shows the comparison of baseline clinical and demographic variables from the original cohort sample (N=345), the present sample, and for subjects who dropped-out during follow-up. These variables did not differ across the three groups.

**Table 1:** Comparison of baseline demographic and clinical variables for the original sample, drop-out sample, and present sample.

Baseline variables	Original sample (N=345)	Drop-out sample (N=111)	Present sample (N=234)	p value
Age (mean±SD)*	70.35 (7.12)	69.69 (7.86)	70.67 (6.74)	0.490
Sex - female (N, %)**	241 (69.9)	81 (73)	160 (68.4)	0.685
Education (mean±SD)*	9.00 (5.47)	9.86 (5.84)	8.59 (5.25)	0.133
Successful agers	214 (62)	71 (64)	143 (61.1)	0.760
Family income (mean±SD)*	22.48 (30.09)	24.88 (35.43)	22.48 (30.07)	0.639
Age of father at death (mean±SD)*	64.50 (16.26)	60.33 (20.00)	64.61 (16.24)	0.904
Age of mother at death (mean±SD)*	73.39 (17.02)	70.25 (2.87)	73.49 (17.29)	0.933
MADRS (mean±SD)*	6.07 (7.37)	5.86 (8.40)	5.77 (7.28)	0.697

* ANOVA analysis of variance;

** Chi-square association test; MADRS (Montgomery-Asberg Depression Rating Scale).

The mortality rate for the whole sample was 41% (N=96). The main causes of death were cardiovascular disease (33%) and cancer (23%), followed by pulmonary disease (17%) and stroke (16%). The mortality rate for successful agers was 32% (N=75) and for normal agers was 55% (N=159).

Table 2 shows the comparison of baseline demographic and clinical data for the groups of deceased and living individuals after 12 years of follow-up. Normal agers at baseline had a higher death rate compared to successful agers (p=0.001).

**Table 2:** Baseline demographic and clinical variables for living versus deceased subjects.

Baseline variables	Deceased (N=96)	Living (N=138)	p value
Age (mean±SD)*	73.32 (7.14)	68.83 (5.80)	0.001
Sex (% female)**	33 (34)	91 (66)	0.002
Monthly family income (mean±SD)*	23.16 (29.49)	20.28 (26.00)	0.447
Successful agers (N, %)**	46 (48)	97 (70)	0.001
Education (mean±SD)*	8.45 (5.38)	8.69 (5.17)	0.738
MADRS (mean±SD)*	6.83 (6.11)	6.31 (5.71)	0.511
Age of father at death (mean±SD)*	62.03 (16.16)	65.70 (16.25)	0.272
Age of mother at death (mean±SD)*	75.83 (16.17)	72.51 (17.74)	0.343
Leisure activities- yes (N, %)**	39 (41)	81 (59)	0.510
Confidant - yes (N, %)**	75(78)	108 (78)	0.897

* Student’s t -test;

** Chi-square association test; MADRS: Montgomery-Asberg Depression Rating Scale.

Higher age, male sex and normal aging were associated with mortality on the univariate Cox regression model (Table 3). All variables entered in the Cox multivariate analysis remained significant in the final model. Normal agers showed 1.8 times higher odds for mortality than successful agers (Table 4).

**Table 3:** Effect of clinical, sociodemographic and aging status on mortality: univariate Cox regression model.

Variables	p	HR	95% CI	
			Lower	Upper
Age	0.001	1.085	1.054	1.118
Sex*	0.002	1.868	1.246	2.801
Aging status**	0.001	2.188	1.464	3.269
Age of mother at death	0.316	1.010	0.990	1.031
Age of father at death	0.546	0.994	0.973	1.014
Leisure activities	0.713	1.086	0.699	1.687
MADRS	0.422	1.013	0.981	1.047
Education	0.812	1.013	0.957	1.035
Family income***	0.478	1.003	0.995	1.010
Confidant (yes)	0.726	1.091	0.672	1.770

* Male sex is reference;

** Normal agers are reference;

*** Family income was measured by number of minimum wages; MADRS: Montgomery Asberg Depression Rating Scale.

**Table 4:** Effect of age, sex and aging status on mortality: multivariate Cox regression model.

Variables	p	HR	95% CI	
			Lower	Upper
Age	<0.001	1.077	1.045	1.109
Sex*	0.002	1.902	1.265	2.858
Aging status **	0.003	1.884	1.464	3.269

* Male sex is reference;

** Normal agers are reference.

Since aging status at baseline (normal or successful) was an independent predictor of mortality, the association of this variable with other outcomes was analyzed. In this cohort, cognitive, functional and clinical status were also measured at each follow-up evaluation, allowing detection of the incidence of dementia, clinical diseases and functional impairment. Normal aging was associated with subsequent dementia (χ^2^=6.99; p=0.008) and functional impairment (χ^2^=5.48; p=0.019) (Pearson chi-square association test). Aging status at baseline was not associated with incident clinical illnesses (p=0.98).

## DISCUSSION

The baseline characteristics of the present cohort of healthy and independent older subjects, with normal or successful aging, provided the opportunity to explore the effect of aging status on mortality. Our main finding was an increased risk of mortality among normal aging subjects (HR=1.88, 95% CI=1.46 to 3.27, p=0.003) adjusted for age and sex. This finding corroborated our initial hypothesis that successful agers have lower mortality rates, and that normal agers have a higher risk for mortality. The concept of successful aging encompasses an exceptional aging process with good state of health, a complete absence of functional disability and mood changes, and no cognitive impairment.^9,10^ Although the aging process is dynamic, and these successful agers will likely experience some kind of decline, this resilience may confer higher longevity. The defining characteristics of successful aging may represent a protective factor for mortality and a trait of lower illness vulnerability. This hypothesis was also corroborated by the finding of a lower frequency of incident dementia and functional impairment among successful agers. Since defining variables of normal or successful aging are potentially modifiable and amenable to intervention, it is relevant to emphasize that mortality rates could be lowered as a consequence of such interventions.

Higher age was a risk factor for mortality in the current investigation. Several previous studies have reported the same result.^2-4^ This result was expected since age is an important determinant of life span, even in a sample of healthy and independent older individuals at baseline. However, it is important to highlight that there is a biological limit to life span determined by chronologic age and independent of trends of increasing life span.^18^

In this study, male sex was also a risk factor for mortality. This finding has previously been reported by the Brazilian statistics for older mortality^19^ and several studies worldwide.^3,5^ Differences in social relationships, health behaviors, functional impairments, socioeconomic status and biological markers observed between sexes have been considered as explanations for this consistent finding across different cultures.^5^

The main causes of death in our cohort were cardiovascular disease and cancer, followed by pulmonary disease and stroke. This finding differs from those observed in the general Brazilian population in whom the leading cause of death is cerebrovascular disease, followed by cardiovascular disease and cancer.^19^ Our results are closer to the findings observed in developed countries, in which cardiovascular disease is the leading cause of mortality.^20^ The PALA study was designed to select healthier older individuals at baseline, composing a sample with similar characteristics to those found in individuals living in more developed countries. Prevention and management of higher determinants of cerebrovascular disease, especially systemic arterial hypertension, are carried out more effectively in these countries, reducing stroke incidence and consequent mortality.^21^

Several limitations of this study should be noted. The 30% drop-out rate during the follow-up might have affected the results. However, as the baseline clinical and demographic variables did not differ among the original cohort, the sample evaluated in this study and the drop-out group, we assumed this sample was representative of the original sample.

The relevance of our investigation was to demonstrate that successful aging impacts mortality. Although this is an expected finding and reflects better aging quality, mortality rates in this aging group have received less attention and our study bridged this gap. It is also important to emphasize the setting in which the study was carried. Data from longitudinal studies with older people residing in less developed countries remain scarce. However, it is relevant to mention some other Brazilian older cohort studies, which have found interesting associations of self-rated health,^22,23^ past medical history, use of health services, dependence in activities of daily living (ADLs), mental health, and cognitive status^23^ with mortality. Finally, our results emphasized the heterogeneity of the healthy aging process and its relevance, indicating that slight differences in the aging process could affect many health outcomes and that this issue warrants further investigation.
